# Increased expression of lncRNA CASC9 promotes tumor progression by suppressing autophagy-mediated cell apoptosis via the AKT/mTOR pathway in oral squamous cell carcinoma

**DOI:** 10.1038/s41419-018-1280-8

**Published:** 2019-01-17

**Authors:** Yixin Yang, Dan Chen, Huan Liu, Kai Yang

**Affiliations:** Department of Oral and Maxillofacial Surgery, The First Affiliated Hospital of Chongqing Medical University, Chongqing, 400016 China

## Abstract

Recent studies showed that lncRNA *CASC9* was upregulated and acted as an oncogene in a variety of tumors. However, the expression and biological functions of *CASC9* in oral squamous cell carcinoma (OSCC) remain unknown. In this study, we found for the first time that *CASC9* was remarkably upregulated in OSCC tissues and cell lines compared with paired noncancerous tissues and normal oral epithelial cells. Highly expressed *CASC9* is strongly associated with tumor size, clinical stage, regional lymph node metastasis and overall survival time in OSCC patients. In vitro, *CASC9* knockdown in OSCC cells SCC15 and CAL27 significantly promotes autophagy and apoptosis, while inhibiting proliferation. Moreover, the expression levels of p-AKT, p-mTOR, P62 and BCL-2 were significantly decreased, while the expression levels of BAX and the LC3BII/LC3BI ratio were increased in *CASC9*-knockdown SCC15 and CAL27 cells. After the addition of the AKT activator SC79 in *CASC9*-knockdown SCC15 and CAL27 cells, we found that the increased autophagy and apoptosis were remarkably rescued. Furthermore, the increased apoptosis was remarkably rescued in *CASC9*-knockdown OSCC cells treated with the autophagy inhibitor Autophinib. In addition, *CASC9* depletion suppressed tumor growth in vivo. In conclusion, our findings demonstrate that lncRNA *CASC9* promotes OSCC progression through enhancing cell proliferation and suppressing autophagy-mediated cell apoptosis via the AKT/mTOR pathway. *CASC9* could potentially be used as a valuable biomarker for OSCC diagnosis and prognosis.

## Introduction

Head and neck cancer is the sixth most common malignant tumor in the world^1^, and oral squamous cell carcinoma (OSCC) is the most common type of head and neck cancer^2^. There are over 300 000 new cases of OSCC every year worldwide, and more than 140 000 patients die of OSCC each year^2,3^. At present, the primary treatment for OSCC is surgery with adjuvant radiation or chemoradiation treatment. Although great progress has been made in surgical techniques, radiation and chemoradiation treatment, the overall 5-year survival rate of OSCC patients has remained approximately 50% for 30 years without any significantly progress^4^. Therefore, further study of the molecular mechanisms underlying OSCC development is the key to developing more effective treatments.

Long noncoding RNA (lncRNAs) is noncoding RNA with a length of more than 200 nt, attracting increasing studies^5^. The number of gene classified as lncRNA is the largest. LncRNA regulates the expression of genes at the level of transcription, posttranscription and translation, affecting various physiological and pathological processes of cells^6–9^. Current studies have shown that variable abnormal expression of lncRNA is closely related to the occurrence of various diseases, including tumors^10–12^. Emerging studies have found that long noncoding RNA cancer susceptibility candidate 9 (*CASC9*) is highly expressed in various cancers, such as esophageal squamous cell carcinoma, nasopharyngeal carcinoma and hepatocellular carcinoma, implying a crucial carcinogenic effect of this lncRNA^13–19^. Although *CASC9* is a lncRNA with extensive clinical prospects, the expression and role of *CASC9* in OSCC remain unclear.

Autophagy is a complex process involving the lysosomal-mediated degradation of intracytoplasmic components. The AKT/mTOR signaling pathway is the primary pathway regulating autophagy^20^, which can determine the survival and death of cells and plays an important role in tumorigenesis^21–23^. Recently, Liang et al. reported that high expression of *CASC9* activates the PI3K/AKT signaling pathway, which promotes the invasion and metastasis of esophageal squamous carcinoma cells^13^. Klingenberg M. et al. demonstrated that increased expression of *CASC9* promotes the phosphorylation of AKT (p-AKT), which induces the proliferation of hepatocellular carcinoma cells^14^. However, it is unclear whether *CASC9* regulates tumor cell autophagy through the AKT/mTOR pathway.

In the present study, we found that *CASC9* is highly expressed in OSCC tissues and cell lines, and the overall survival time of patients with higher levels of *CASC9* expression is significantly shorter compared with patients with low expression. Moreover, silencing *CASC9* inhibits OSCC growth in vivo. More importantly, we discovered for the first time that *CASC9* regulates autophagy through the AKT/mTOR pathway in tumor cells, promoting autophagy-mediated apoptosis.

## Results

### *CASC9* is increased in OSCC tissues and cell lines

**Fig. 1:**
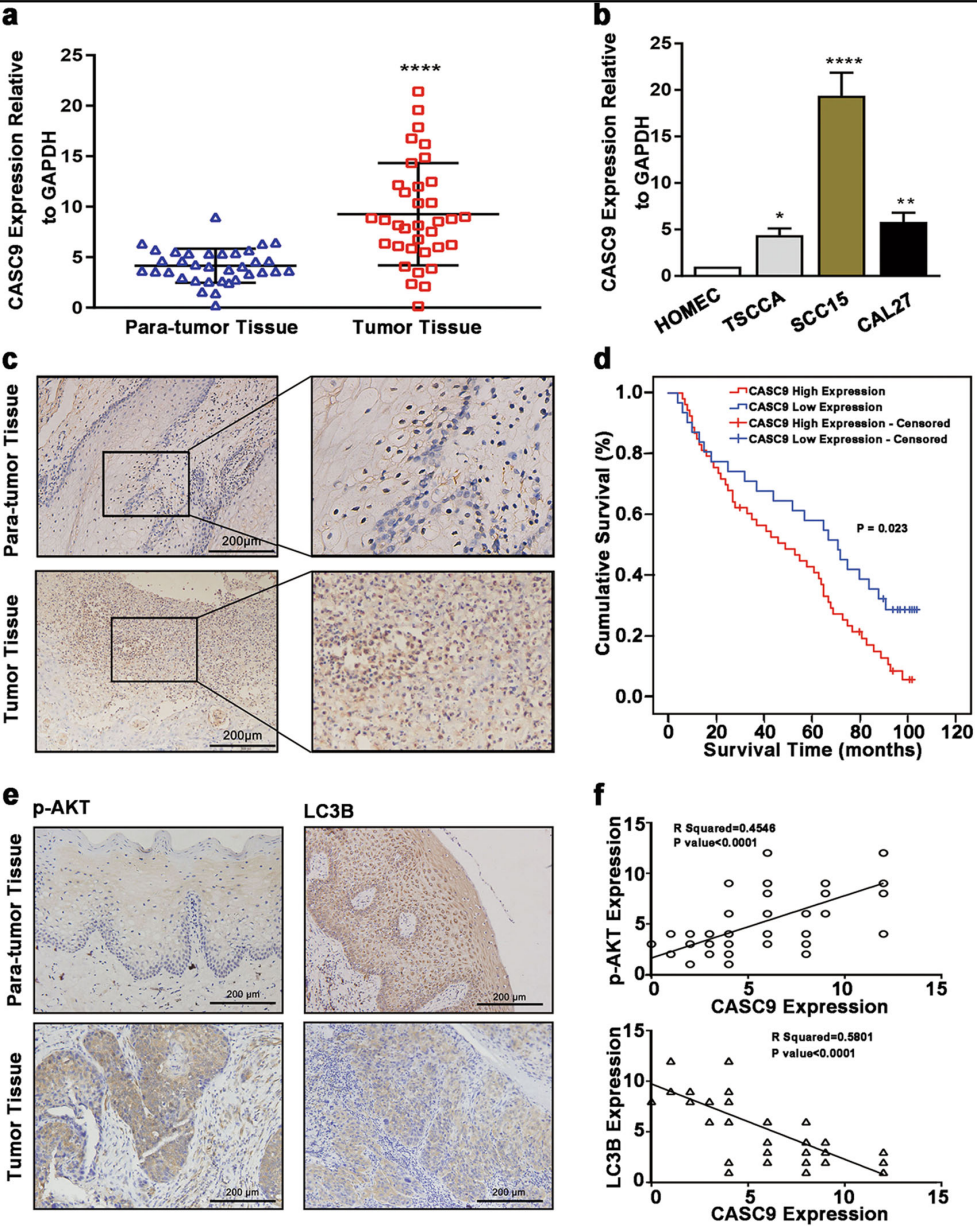
*CASC9* is highly expressed in OSCC tissues and cells. **a** RT-qPCR results showed that *CASC9* expression was significantly increased in OSCC tissues compared with paired adjacent tissues (*N* = 35). **b** RT-qPCR results showed that *CASC9* expression was significantly increased in TSCCA, SCC15 and CAL27 OSCC cells compared to the normal oral mucosal HOMEC cells. **c** The ISH results showed that the expression level of *CASC9* in OSCC tissues was significantly higher compared with the paired adjacent tissues (*N* = 84; scale bars = 200 μm). **d** The mean overall survival time of OSCC patients with a high expression level of *CASC9* was significantly lower compared with patients with a low expression level. **e** IHC analysis showed that the expression of p-AKT was significantly increased in OSCC tissues compared with matched para-carcinoma tissues, and the expression level of LC3 B in OSCC tissues was significantly decreased (*N* = 84; scale bars = 200 μm). **f** *CASC9* expression levels are positively correlated with p-AKT and negatively correlated with LC3B in OSCC tissues (*N* = 84). All data represent three independent experiments. Data are presented as the mean ± SD (n ≥ 3). *P < 0.05, **P < 0.01, ***P *<* 0.001, ****P *<* 0.0001

RT-qPCR was performed to analyze *CASC9* expression in 35 cases of OSCC tissues and paired para-tumor tissues. The results revealed that the expression of *CASC9* in OSCC tissues was significantly higher compared with adjacent normal tissues (*P* *<* 0.0001) (Fig. 1a). The expression of *CASC9* in normal oral mucosal cell HOMEC cell and oral squamous cell carcinoma cells, including TSCCA, SCC15 and CAL27 was further detected by RT-qPCR. Similarly, the results revealed that *CASC9* expression levels in TSCCA, SCC15 and CAL27 cells were significantly higher compared with HOMEC cells (*P* *<* 0.05) (Fig. 1b). These findings demonstrate that *CASC9* expression is significantly elevated in OSCC.

### Elevated *CASC9* is related to poor prognosis in OSCC patients

To verify the relationships between elevated *CASC9* expression and prognosis in OSCC patients, ISH was performed to detect the expression of *CASC9* in 84 cases of OSCC patients with complete clinical data and follow-up data (cohort 2), and the relationship between *CASC9* expression and clinicopathological features and overall survival time were analyzed in cohort 2. Consistent with the results of cohort 1, the expression of *CASC9* in OSCC was higher compared with the corresponding adjacent tissues (*P* *<* 0.05) (Fig. 1c and Table 1). *CASC9* was located both in the nucleus and cytoplasm in OSCC, and *CASC9* was distributed primarily in the cytoplasm (Fig. 1c). What’s more, *CASC9* expression was found to be significantly correlated with tumor size, regional lymph node metastasis and clinical stage of OSCC (*P* *<* 0.05). Kaplan–Meier survival analysis showed that the mean overall survival time of patients with high and low *CASC9* expression was 49.3 ± 4.3 months and 62.9 ± 6.5, months, respectively, which means that the overall survival time of OSCC patients with elevated *CASC9* expression was significantly shorter than that of patients with low expression (*P* *=* 0.023) (Fig. 1d). Multivariate Cox regression analysis revealed that the *CASC9* expression level was an independent prognostic factor in OSCC patients (Table 2). IHC revealed that p-AKT was highly expressed in OSCC relative to the corresponding adjacent normal tissues and positively correlated with *CASC9* expression (*R*^2^ = 0.4546, *P* *<* 0.001). LC3B was downregulated in OSCC relative to adjacent normal tissues and negatively correlated with *CASC9* expression (*R*^2^ = 0.5801, *P* *<* 0.001) (Fig. 1e, f). These results indicate that *CASC9* is an independent prognostic factor affecting the survival of OSCC patients. Moreover, elevated *CASC9* expression in OSCC may play a role in promoting cancer by activating the AKT/mTOR signaling pathway to regulate autophagy.

**Table 1:** The expression of CASC9 and its relationship with clinicopathological features of patients with OSCC

Parameters	Total	CASC9 Expression		χ^2^	*P* value
		High	Low		
Tissue type				9.47	0.002*
OSCC	84	53	31		
ANT	32	10	22		
Age				2.647	0.266
≥60	38	27	11		
40–60	35	21	14		
<40	11	5	6		
Gender				0.568	0.451
Male	47	28	19		
Female	37	25	12		
Tumor differentiation				2.849	0.241
Well	28	20	8		
Moderate	31	16	15		
Poor	25	17	8		
T staging				9.31	0.025*
T1	14	5	9		
T2	21	11	10		
T3	32	23	9		
T4	17	14	3		
Regional lymph node metastasis				10.063	0.002*
No	49	24	25		
Yes	35	29	6		
Clinical stage				14.227	0.003*
I	12	4	8		
II	14	5	9		
III	38	27	11		
IV	20	17	3		
Site				5.883	0.208
Gingiva	13	10	3		
Tongue	33	24	9		
Buccal	26	14	12		
The floor of the oral	8	3	5		
Palate	4	2	2		

*P* values reflect the relationship between CASC9 expression and clinicopathological parameters with Chi-square test. *P* *<* 0.05 was considered statistically significant. *P < 0.05. High (Score 6–12), Low (Score 0–4)

*OSCC* Oral squamous cell carcinoma, *ANT* adjacent noncancerous tissues

**Table 2:** Univariate analysis and multivariate analysis of various progression in patients with OSCC Cox-regression analysis

	Univariate analysis			Multivariate analysis		
	*P* value	HR	95% CI	*P* value	HR	95% CI
CASC9	0.025*	0.558	0.335–0.930	0.023*	2.31	1.123–4.752
Age	0.758	0.951	0.693–1.306	0.832	1.045	0.695–1.572
Gender	0.652	0.897	0.561–1.437	0.467	0.821	0.482–1.398
Tumor differentiation	0.277	1.18	0.875–1.592	0.317	1.193	0.844–1.687
T classification	< 0.001*	6.36	4.232–9.559	< 0.001*	3.711	2.077–6.631
Regional lymph node metastasis	< 0.001*	7.832	4.274–14.351	0.006*	2.749	1.340–5.643
Clinical stage	< 0.001*	5.092	3.439–7.539	0.011*	2.383	1.224–4.637
Site	0.902	0.984	0.767–1.263	0.924	0.987	0.761–1.282

*HR* hazard ratio *CI* confidence interval

*P < 0.05

### Suppression of *CASC9* inhibits proliferation and promotes tumor cell apoptosis

**Fig. 2:**
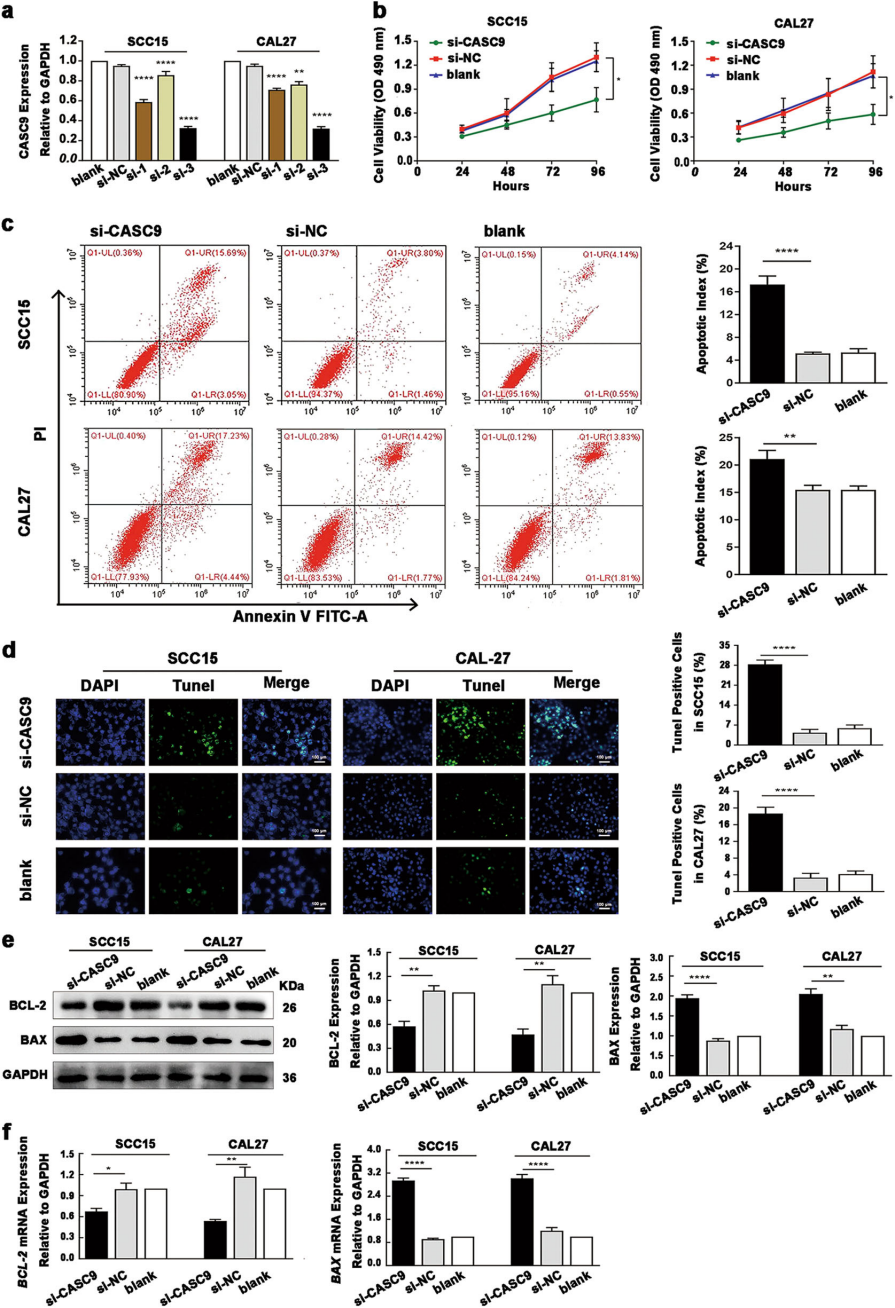
*CASC9* depletion inhibits the proliferation of SCC15 and CAL27 cells and promotes apoptosis. **a** The verification of the knockdown efficiency of three siRNAs (si-1, si-2, si-3) targeting different sites of *CASC9* in SCC15 and CAL27 cells showed that si-3 was the most effective siRNA for silencing *CASC9* in both SCC15 and CAL27 cells. **b** The MTT assay revealed that the proliferation of *CASC9*-knockdown SCC15 and CAL27 cells was significantly increased. **c** Flow cytometric analysis revealed that the apoptosis index of *CASC9*-knockdown SCC15 and CAL27 cells increased significantly. **d** The TUNEL assay showed that the rate of TUNEL-positive cells in SCC15 and CAL27 cells increased significantly after the expression of *CASC9* was silenced. **e**, **f** Western blotting (**e**) and RT-qPCR assay (**f**) showed that the expression level of *BCL-2* was significantly reduced after *CASC9* was silenced, and the *BAX* expression level was significantly increased. All data represent three independent experiments. Data are presented as the mean ± SD (*n* ≥ 3). *P < 0.05; **P < 0.01; ***P *<* 0.001; ****P *<* 0.0001

To explore the effect of altered *CASC9* expression on OSCC cells, SCC15 and CAL27 cells, in which *CASC9* is the most highly expressed, were selected for the following assays. We verified the knockdown efficiency of three siRNAs (si-1, si-2, si-3) targeting different sites of *CASC9* in SCC15 and CAL27 cells, and the results showed that si-3 was the most effective siRNA for silencing *CASC9* in both SCC15 and CAL27 cells (*P* *<* 0.0001) (Fig. 2a). Therefore, si-3 was selected for all subsequent experiments. Three experimental groups were defined, as follows: the experimental group (si-*CASC9*), negative control group (si-NC), and blank control group (blank).

To explore the effects of altered *CASC9* expression on the proliferation and apoptosis of OSCC cells, MTT assay, flow cytometry, and TUNEL assays were performed. The MTT assay revealed that the proliferation of SCC15 and CAL27 cells was significantly decreased after *CASC9* expression was silenced (*P* *<* 0.05) (Fig. 2b). In addition, the apoptotic index of SCC15 and CAL27 was significantly increased after *CASC9* expression was silenced, as detected by flow cytometry (*P* *<* 0.05) (Fig. 2c). The TUNEL assay showed that the apoptotic rate of SCC15 and CAL27 cells was significantly increased after *CASC9* expression was silenced (Fig. 2d). Western blotting and RT-qPCR analysis revealed that the protein and mRNA expression levels of *BCL-2* were significantly decreased (*P* *<* 0.05), while the protein and mRNA expression levels of *BAX* were significantly increased in the *CASC9*-knockdown SCC15 and CAL27 cells (*P* *<* 0.05) (Fig. 2e, f). These findings demonstrate that the cell proliferation was inhibited, while the apoptosis was enhanced in *CASC9*-knockdown SCC15 and CAL27 cells.

### Depletion of *CASC9* triggers autophagy in OSCC cells

**Fig. 3:**
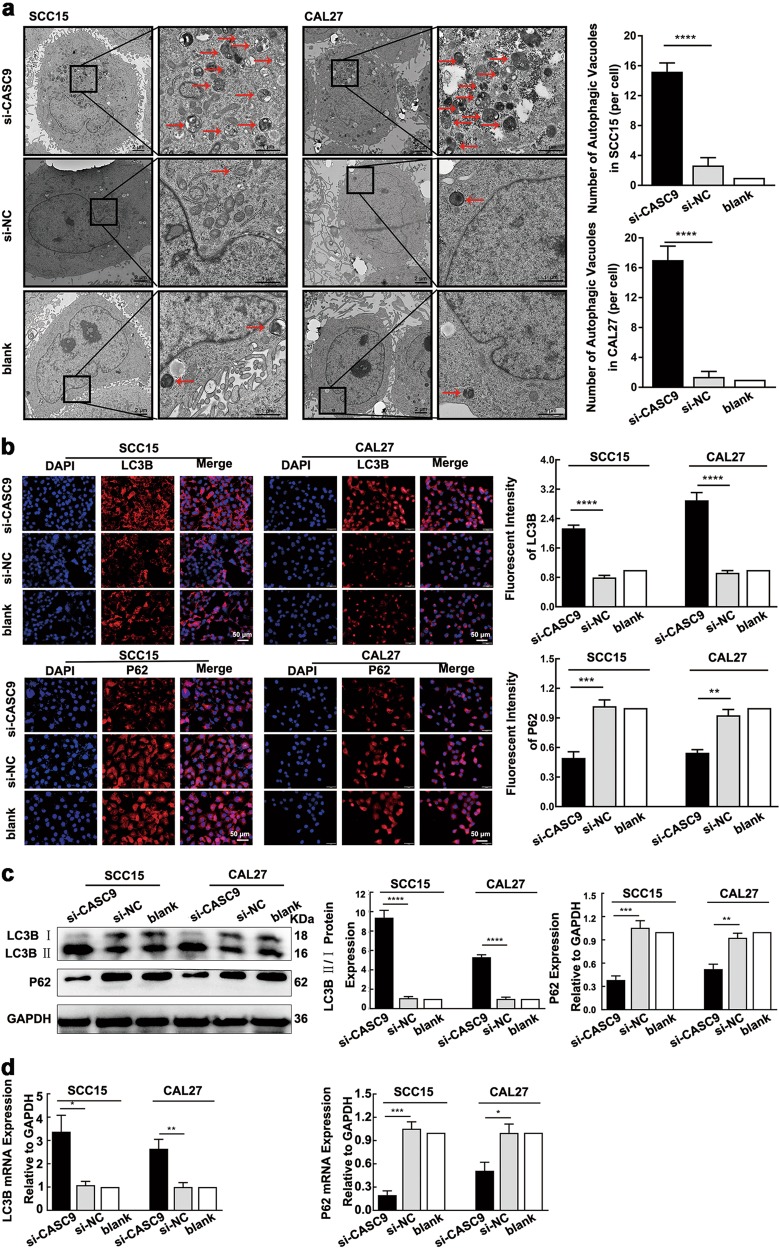
*CASC9* knockdown promotes autophagy in SCC15 and CAL27 cells. **a** TEM revealed an increase in the density of autophagosomes in SCC15 and CAL27 cells after *CASC9* was silenced. (low magnification scale bars = 2 μm; high magnification scale bars = 1 μm). **b** The immunofluorescence assay showed that the fluorescence intensity of LC3B was enhanced in *CASC9*-silenced SCC15 and CAL27 cells, whereas the fluorescence intensity of P62 was reduced (scale bars = 200 μm). **c**, **d** After the knockdown of *CASC9* in SCC15 and CAL27 cells, the LC3B II/LC3B I ratio was increased, as detected by western blotting, and the expression level of P62 protein was decreased (**c**); likewise, the mRNA expression of *LC3B* was increased, and that of *P62* was decreased (**d**). All data represent three independent experiments. Data are presented as the mean ± SD (n ≥ 3). *P < 0.05; **P < 0.01; ***P *<* 0.001; ****P *<* 0.0001

Autophagic density was significantly increased in SCC15 and CAL27 cells after the knockdown of *CASC9*, according to the TEM analysis (*P* *<* 0.001) (Fig. 3a). The immunofluorescence assay showed that the fluorescence intensity of the LC3B protein in *CASC9*-knockdown SCC15 and CAL27 cells was significantly increased (*P* *<* 0.001), while the fluorescence intensity of the P62 protein was significantly decreased (*P* *=* 0.001) (Fig. 3b). After *CASC9* expression was silenced, the LC3B II/LC3B I ratio was significantly increased (*P* *<* 0.05), while the P62 expression level was significantly decreased (*P* *<* 0.05) (Fig. 3c), as detected by western blotting. *LC3B* mRNA expression was significantly elevated (*P* < 0.05), while *P62* mRNA expression was significantly reduced (*P* < 0.05) (Fig. 3d), as detected by RT-qPCR in *CASC9*-knockdown SCC15 and CAL27 cells. These findings demonstrate that the depletion of *CASC9* triggers autophagy in SCC15 and CAL27 cells.

### *CASC9* knockdown reduces the AKT/mTOR signaling pathway activation in OSCC cells

**Fig. 4:**
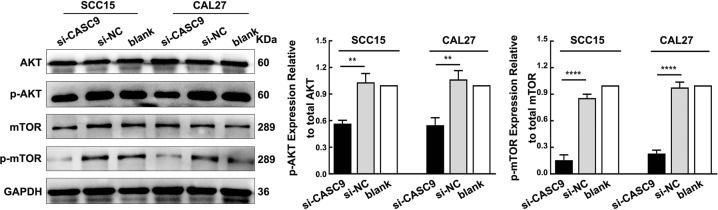
Silencing *CASC9* inhibits the activation of the AKT/mTOR signaling pathway. The expression levels of p-AKT and p-mTOR in SCC15 and CAL27 cells were significantly decreased, as detected by western blotting, while the protein expression levels of total AKT and total mTOR were not significantly altered. All data represent three independent experiments. Data are presented as the mean ± SD (n ≥ 3). *P < 0.05; **P < 0.01; ***P *<* 0.001; ****P *<* 0.0001

Emerging evidence suggests that *CASC9* regulates the AKT signaling pathway in esophageal squamous cell carcinoma and hepatocellular carcinoma^13,14^. To investigate whether *CASC9* affects the AKT/mTOR signaling pathway, the expression of p-AKT and p-mTOR, which are key proteins in the AKT/mTOR pathway, was examined. Western blotting analysis revealed that there was no significant change in the total AKT or total mTOR expression after *CASC9* was silenced in SCC15 and CAL27 cells (*P* *>* 0.05), whereas the expression of p-AKT and p-mTOR was significantly decreased (*P* *<* 0.05) (Fig. 4). These results demonstrate that *CASC9* knockdown reduces the activation of the AKT/mTOR signaling pathway in OSCC cells.

### *CASC9* inhibits apoptosis and autophagy through the AKT/mTOR signaling pathway in OSCC cells

**Fig. 5:**
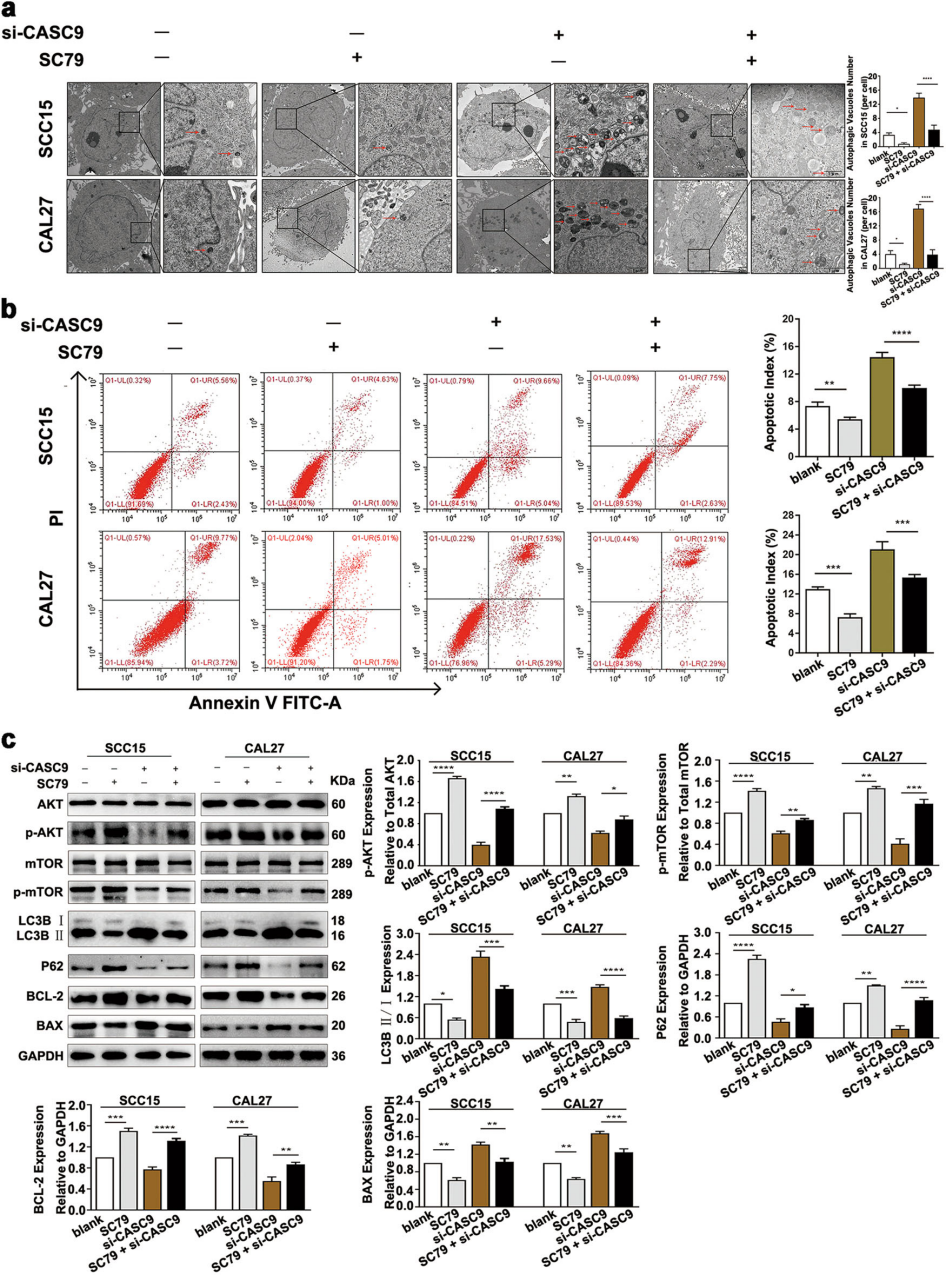
*CASC9* inhibits apoptosis and autophagy in OSCC cells through the regulation of the AKT/mTOR signaling pathway. **a** TEM experiments revealed that the autophagosome density was significantly reduced after the addition of the AKT activator SC79 to *CASC9*-knockdown SCC15 and CAL27 cells (low magnification scale bars = 2 μm; high magnification scale bars = 1 μm). **b** Flow cytometry revealed that after the addition of the AKT activator SC79 to *CASC9*-knockdown SCC15 and CAL27 cells, the apoptotic index was significantly reduced. **c** Western blotting revealed that the expression of p-mTOR was significantly increased after the activation of the AKT signaling pathway by the addition of SC79 to *CASC9*-knockdown SCC15 and CAL27 cells; moreover, the LC3B II/LC3B I ratio was significantly decreased, P62 protein expression was partially elevated, BCL-2 protein expression was partially increased, and BAX protein expression was partially reduced. Blank means blank control group, which was treated with no SC79 or si-CASC9. All data represent three independent experiments. Data are presented as the mean ± SD (*n* ≥ 3). *P < 0.05; **P < 0.01; ***P *<* 0.001; ****P *<* 0.0001

The AKT/mTOR signaling pathway is important for regulating cell autophagy and apoptosis^24,25^. To investigate whether *CASC9* regulates autophagy and apoptosis in OSCC through the regulation of the AKT/mTOR signaling pathway, autophagy and apoptosis were measured after the addition of an AKT activator (SC79) (HY-18749, MCE, New Jersey, USA) in *CASC9*-knockdown SCC15 and CAL27 cells. The results showed that treatment with SC79 significantly reduced the autophagosome density and apoptotic index (Fig. 5a, b) compared to the controls. Moreover, the LC3B II/LC3B I ratio and BAX expression were significantly reduced, whereas BCL-2 expression was significantly increased (Fig. 5c). These results demonstrate that adding SC79 into transfected SCC15 and CAL27 cells alleviated the *CASC9*-knockdown mediated increase in autophagy and apoptosis. In summary, *CASC9* regulates autophagy and apoptosis through the regulation of the AKT/mTOR signaling pathway in OSCC.

### *CASC9* reduces apoptosis by inhibition of autophagy in OSCC cells

**Fig. 6:**
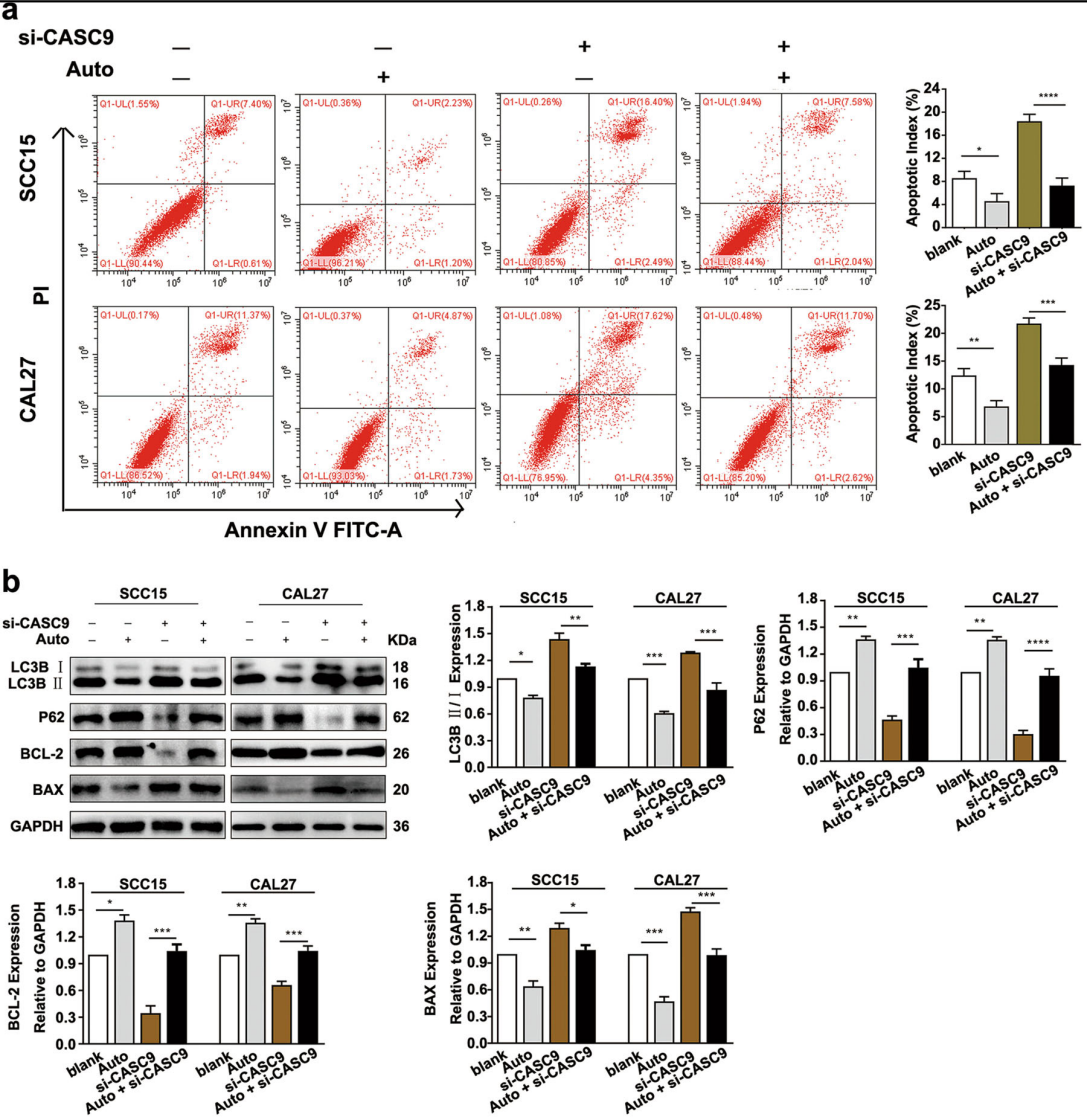
*CASC9* regulated apoptosis depending on autophagy in OSCC cells. **a** Flow cytometry revealed that after the addition of the autophagy inhibitor Autophinib (Auto) to *CASC9*-knockdown SCC15 and CAL27 cells, the apoptotic index was significantly decreased. **b** Western blotting revealed that the addition of Autophinib to *CASC9*-knockdown SCC15 and CAL27 cells increased the protein expression of BCL-2 and decreased the protein expression of BAX. Blank means blank control group, which was treated with no Autophinib or si-CASC9. All data represent three independent experiments. Data are presented as the mean ± SD (*n* ≥ 3). *P < 0.05; **P < 0.01; ***P *<* 0.001; ****P *<* 0.0001

To investigate the regulatory relationship between autophagy and apoptosis in OSCC, we detected apoptosis in SCC15 and CAL27 cells that were cotreated with si-*CASC9* and the autophagy inhibitor (Autophinib) (HY-101920, MCE, New Jersey, USA). Flow cytometric analysis revealed that the apoptotic index of cells coincubated with si-*CASC9* and Autophinib was significantly decreased compared to cells without Autophinib treatment (*P* *<* 0.05) (Fig. 6a). Western Blotting analysis further revealed that, in the cells cotreated with si-*CASC9* and Autophinib, BCL-2 protein expression was significantly increased, while BAX protein expression was significantly decreased compared to the cells treated only with si-*CASC9* (*P* *<* 0.05) (Fig. 6b). These results demonstrate that inhibition of autophagy in OSCC cells reversed the increased rate of apoptosis caused by the *CASC9* knockdown, suggesting that the regulation of apoptosis by *CASC9* in OSCC is dependent on the regulation of autophagy.

### *CASC9* knockdown suppressed tumor growth in vivo

**Fig. 7:**
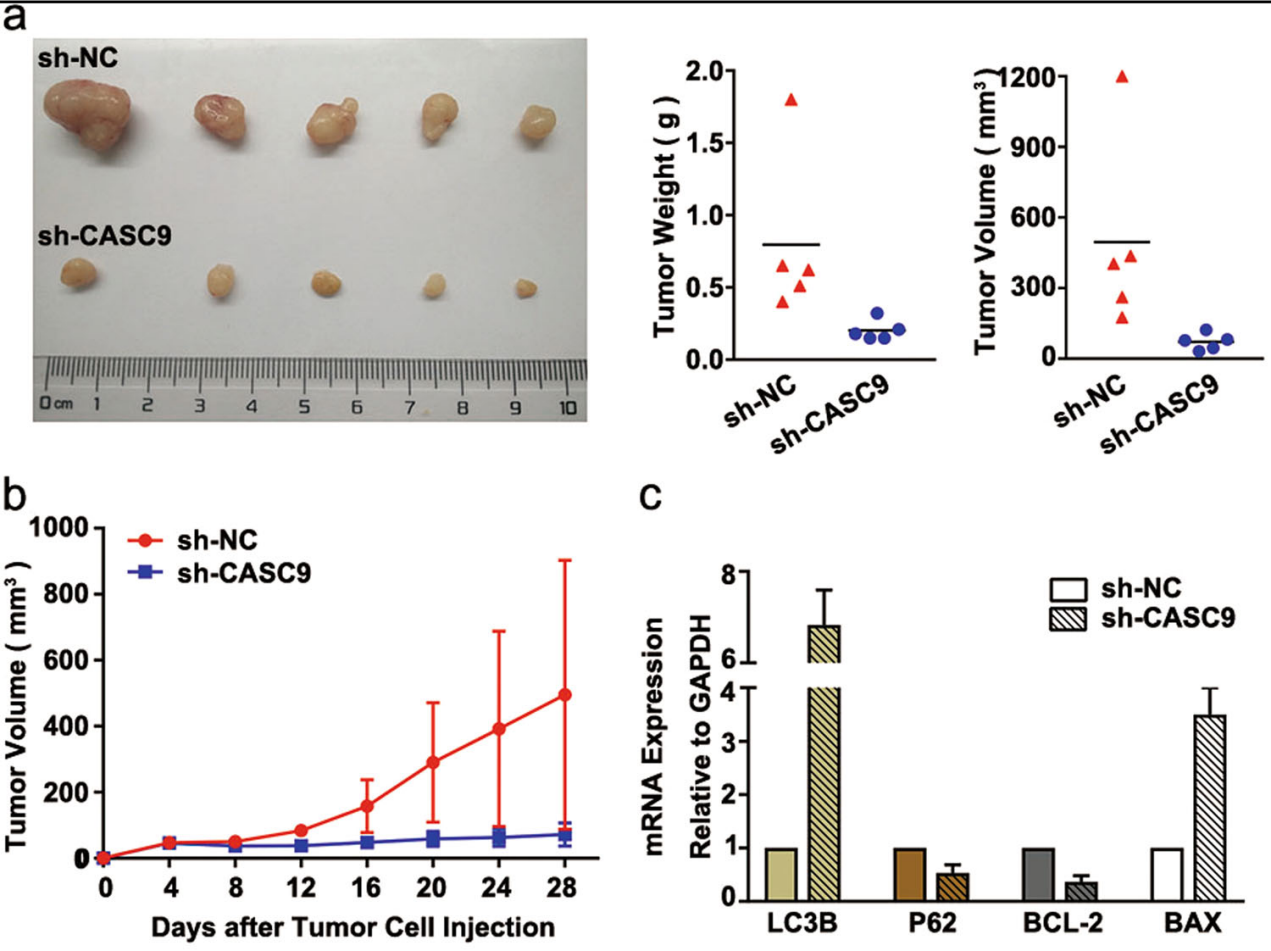
Silencing *CASC9* inhibits the tumorigenic ability of OSCC cells in vivo. **a** The weight and volume of tumors in the sh-*CASC9* group were significantly less than those in the sh-NC group (*N* = 5). **b** The growth curve of the in vivo tumorigenicity assay showed that the subcutaneous tumor growth of the sh-*CASC9* group was significantly slower. **c** RT-qPCR was used to detect tumor-forming tissues in vivo. The results showed that the mRNA expression level of *LC3B* was decreased and the mRNA level of *P62* was decreased in the sh-*CASC9* group; the mRNA expression level of *BCL-2* was decreased and the mRNA expression level of *BAX* was increased in sh-*CASC9* group. All data represent three independent experiments. Data are presented as the mean ± SD (*n* ≥ 3). *P < 0.05; **P < 0.01; ***P *<* 0.001; ****P *<* 0.0001

An in vivo tumorigenicity assay in nude mice revealed that the weight and volume of tumors in the sh-*CASC9* group were significantly less than those in the sh-NC group (Fig. 7a). Meanwhile, the tumor growth of the sh-*CASC9* group was slower compared to that of the sh-NC group (*P* *<* 0.05) (Fig. 7b). RT-qPCR analysis revealed that, compared with the sh-NC group, the mRNA expression level of *LC3B* and *BAX* mRNA was increased in the sh-*CASC9* tumor tissues, whereas the mRNA expression level of *P62* and *BCL-2* was decreased (*P* *<* 0.05) (Fig. 7c). These results indicate that silencing *CASC9* significantly inhibits the growth of tumors in vivo.

## Discussion

Less than 2% of the RNA produced by transcription of the human genome encodes for proteins, and nearly 98% of the RNA does not encode for proteins, the latter is called noncoding RNA (ncRNA)^26,27^. Over 68% of genes were classified as lncRNAs^28^. Previously, lncRNA was considered to be nonfunctional “noise.” However, in recent years, extensive research has demonstrated that abnormally expressed lncRNA is closely related to the occurrence and development of various cancers. In addition, lncRNA is regarded as an important marker for cancer diagnosis and prognosis as well as a therapeutic target for cancer treatment^29–36^. lncRNA is characterized by strong tissue specificity, and its expression level and function may vary greatly between different cell types^37^. However, recent studies have determined that *CASC9* is highly expressed in many cancers, such as esophageal squamous cell carcinoma, nasopharyngeal carcinoma and hepatocellular carcinoma. Moreover, this increased expression is significantly correlated with tumor size, clinical stage and overall survival time, which indicates that *CASC9* plays an important carcinogenic role in various cancers^13–19^. The present study also demonstrated that increased *CASC9* in OSCC is significantly associated with tumor size, regional lymph node metastasis, clinical stage, and overall survival time, suggesting that *CASC9* may be an effective therapeutic target and prognostic marker in a variety of tumors. Therefore, there are broad and important clinical prospects of *CASC9*, which warrant further research.

Disorder of cell proliferation, apoptosis, and metastasis is an important trigger of cancer occurrence and development. The PI3K/AKT signaling pathway is an important signaling pathway that regulates cell proliferation, apoptosis, and metastasis^38–40^. Recent studies have shown that autophagy plays an important role in tumorigenesis^21–23^. Moreover, mTOR, which is downstream of the PI3K/AKT signaling pathway, is a crucial negative regulator of autophagy^25^. Studies on esophageal squamous cell carcinoma and hepatocellular carcinoma have shown that high expression of *CASC9* activates the PI3K/AKT signaling pathway, promoting the proliferation, invasion and metastasis of cancer cells^13,14^. The present study also demonstrated that increased *CASC9* expression promotes OSCC cell proliferation. It is unclear whether the increased *CASC9* expression in cancer cells regulates the expression of mTOR through the regulation of the PI3K/AKT signaling pathway to control autophagy. In this study, depletion of *CASC9* in OSCC cells led to the significantly decreased expression of p-AKT and p-mTOR, as well as increased autophagy. Treatment with an AKT activator in *CASC9*-knockdown cells partially rescued the decreased p-mTOR expression and increased autophagy. In addition, the expression of *CASC9* was also positively correlated with p-AKT and negatively correlated with LC3B in OSCC tissue samples. Taken together, these findings demonstrate for the first time that *CASC9*, which is highly expressed in cancer cells, inhibits autophagy by activating the AKT/mTOR pathway.

Autophagy plays an important role in the occurrence and development of tumors^22,23,41–43^. There exists crosstalk between autophagy and apoptosis; autophagy can both inhibit and promote apoptosis to affect the occurrence and development of cancers^21^. Due to the diversity of cells, conditions and stimulating factors, autophagy acts as a double-edged sword for apoptosis^44^. For example, Zhao Z. et al. found that oxamate inhibits apoptosis by promoting autophagy to promote gastric cancer;^45^ in contrast, Yeh P.S. et al found that honokiol can promote apoptosis by promoting autophagy to promote neuroblastoma^46^. Our study found that autophagy and apoptosis were both increased in cells with silenced *CASC9*. However, apoptosis was significantly reduced in the cells cotreated with si-*CASC9* and the autophagy inhibitor, indicating that silencing *CASC9* induced autophagy as a pro-death response. The current study found that autophagy can regulate early apoptosis^47,48^. For example, Fan et al.^48^ found resveratrol-induced autophagy enhances early apoptosis in HL-60 cells. This study also found that *CASC9* knockdown in OSCC cells increased the early apoptosis. It is shown that P62 can regulate both autophagy and apoptosis, and many studies reported that P62 mediates apoptosis primarily through regulating autophagy^49^. Bjørkøy G. et al^50^ reported that P62 is incorporated into autophagosomes through binding to LC3 in autophagy-activated cells, and subsequently P62 is degraded by autophagy; thus, P62 protein expression decreases, and reduced P62 protein expression increases cell apoptosis. In this study, autophagy and apoptosis were enhanced in *CASC9*-knockdown OSCC cells, and P62 protein expression was decreased. Moreover, rescue experiments showed that autophagy positively regulates apoptosis. Thus, we supposed that *CASC9* depletion in OSCC cells might promote the binding of P62 to LC3, and then P62 is incorporated into autophagosomes, resulting in the degradation of P62; subsequently, reduction of P62 may increase the autophagy-mediated apoptosis. However, the detailed mechanism remains to be further studied.

**Fig. 8:**
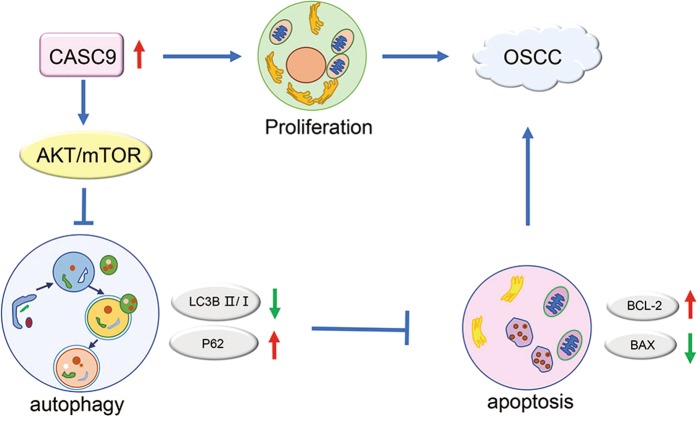
Schematic of oncogenic role of *CASC9* in OSCC. *CASC9* promotes OSCC progression through enhancing cell proliferation and suppressing autophagy-mediated cell apoptosis via activating the AKT/mTOR signaling pathway

In summary, this study found for the first time that increased *CASC9* was significantly associated with tumor size, regional lymph node metastasis, clinical stage and overall survival time in OSCC patients. Experiments in vivo and in vitro demonstrated that high expression of *CASC9* promotes the proliferation of OSCC cells. More importantly, we found for the first time that *CASC9* regulates autophagy in tumor cells. We also demonstrated that *CASC9* depletion increases autophagy by inhibiting the AKT/mTOR signaling pathway to promote autophagic apoptosis in OSCC cells (Fig. 8). These findings suggest that *CASC9* could potentially be used as a valuable biomarker for OSCC diagnosis and prognosis.

## Materials and Methods

### Human tissue samples

There were two groups of OSCC patients tissue samples used. Cohort 1 included fresh OSCC tissue and matched adjacent normal tissues from 35 patients who underwent surgical resection in the oral and maxillofacial surgery department of the first hospital affiliated to Chongqing Medical University between July 2017 and November 2017. The fresh tissues were stored in liquid nitrogen immediately after resection. Cohort 2 included paraffin-embedded tissue sections of 84 OSCC patients obtained from the department of pathology, the First Affiliated Hospital of Chongqing Medical University. Patients in cohort 2 were hospitalized in the oral and maxillofacial surgery department of the first hospital affiliated to Chongqing Medical University between January 2007 and February 2011. The clinicopathological data of all patients are shown in Table 1. Thirty-two para-cancerous tissues were selected from cohort 2 as a control group. All of the patients included in the study were diagnosed by pathology, and no treatment, such as radiotherapy or chemotherapy, was performed before the operation. The study was approved by the Biomedical Ethics Committee of the First Affiliated Hospital of Chongqing Medical University (ApprovaI number: 2017–053), and all patients provided informed consent.

### Cell culture and reagents

Normal oral mucosal HOMEC cells were purchased from Shanghai Bioleaf Biotechnology Co., Ltd. (Shanghai, China); OSCC TSCCA cells were purchased from Shanghai Zhong Qiao Xin Zhou Biotechnology Co., Ltd. (Shanghai, China); OSCC SCC15 and CAL27 cells were donated by Professor Huang Enyi from Chongqing Key Laboratory of Oral Diseases and Biomedicine. The cells were cultured in Dulbecco’s modified Eagle’s medium (DMEM, Gibico, USA) containing 10% fetal bovine serum (FBS) and 1% penicillin-streptomycin in an incubator containing 5% CO_2_ at 37 °C.

### In situ hybridization (ISH)

In situ hybridization (ISH) was performed using a kit (MK10506-h, Boster, Wuhan, China) according to the manufacturer’s protocol. Briefly, a hybridization probe specific for *CASC9* in ISH was designed according to 3 transcripts of *CASC9* in NCBI (https://www.ncbi.nlm.nih.gov/). The probe sequences are shown in Supplementary Table. S1. The probes were synthesized by Boster bioengineering co., LTD. (Boster, Wuhan, China). Paraffin-embedded OSCC and matched adjacent tissue sections were digested at room temperature for 2 min with fresh 3% citrate diluted pepsin (1 ml of 3% citrate plus 2 drops of condensed pepsin). Next, the sections were deparaffinized and rehydrated, washed with PBS 3 times for 5 min and then washed with distilled water, and then incubated with 20 μl of preliminary hybrid liquid at 37 °C for 2 h. Then, the slides were subsequently incubated with 20 μl of hybrid liquid at 42 °C overnight. For the negative control, the hybrid liquid was replaced with preliminary hybrid liquid. After hybridization, the slides were washed in gradient dilute solution SSC buffer, incubated with biotin-labeled rat anti-digoxin for 60 min at 37 °C, and then washed with PBS 4 times. Each slide was developed with DAB and observed under the optical microscope. Tissues were counterstained with Mayer’s Hematoxylin, differentiated with 0.1% alcohol hydrochloride, and washed with running water. Subsequently, the slides were dehydrated and then sealed with neutral gum. Images were captured with the fluorescence microscope (BX51TRF, OLYMPUS, Japan), and a brown color was considered positive. The results were evaluated by double grading and semiquantitative grading.

### Immunohistochemistry (IHC)

Immunohistochemistry was performed using a kit (Beijing, Zhong Shan Jin Qiao, SP-9000) according to the manufacturer’s protocol. In brief, 4-μm paraffin-embedded sections were routinely deparaffinized and rehydrated. Antigen retrieval was accomplished by microwaving the sections in 10 mM citrate buffer (pH 6.0). The activity of endogenous peroxidase was eliminated with 3% H_2_O_2_. Then, the slides were incubated with normal goat serum seal solution at room temperature for 20 min to prevent nonspecific binding. Next, the slides were incubated with primary antibodies against p-AKT and LC3B overnight at 4 °C, and then the slides were incubated with biotin-labeled goat anti-mouse/rabbit IgG secondary antibodies at room temperature for 10 min. Streptavidin-peroxidase was applied for 15 min at room temperature followed by development with DAB, and then the sections were counterstained with Mayer’s Hematoxylin for 2 min. Subsequently, the sections were dehydrated and sealed with neutral gum. For the negative control, PBS was used in place of the primary antibody. The results were evaluated by double grading and semiquantitative grading.

### Vector construction and transfection

Three siRNAs (si-1, si-2, si-3) were designed according to the common sequence of *CASC9* transcripts in NCBI (https://www.ncbi.nlm.nih.gov/), with a nonspecific scramble siRNA as a negative control (si-NC). The siRNAs were designed and synthesized by Shanghai GenePharma Co., Ltd. (Shanghai, China), and the sequences are listed in Supplementary Table S2. Briefly, cells at a concentration of 5 × 10^4^/ml were incubated for 24 h in six-well plates in culture medium. When the cell density reached 50–70%, the cells were transfected with *CASC9* siRNA and si-NC in the presence of Lipofectamine 3000 (Invitrogen, USA). After transfection for 48 h, RNA was collected for quantitative real-time polymerase chain reaction (RT-qPCR) to verify the knockdown efficiency. The sequences of sh-*CASC9* and sh-NC were designed according to the sequences of si-3 and si-NC, respectively as follows: CCGG was added at the 5’ end of the sequence, while TTTTTG was added at the 3’ end of the sequence. Then, a CTCGAG loop was added in the middle, and Age I and EcoRI restriction sequences were added to both ends. The sh-RNAs were synthesized and packaged in lentiviral vectors by Shanghai GenePharma Co., Ltd. (Shanghai, China), designated sh-*CASC9* and sh-NC. The sequences are shown in Supplementary Table. S2. During logarithmic growth, SCC15 cells were seeded into 4 ml of DMEM/F12 medium containing 10% FBS to which 1 ml of medium containing the lentiviral vectors and 5 μl of Polybrene were added. The cells were cultured for 24 h in an incubator at 37 °C under an atmosphere of 5% CO_2_. Fresh culture medium containing 2 μg/ml puromycin was added once per day. Stable pools were obtained 7 days later.

### Immunofuorescence assay

Cell lines were seeded on glass coverslips. After incubation in 100% methanol (chilled at −20 °C) at room temperature for 5 min, the cells were washed three times with ice-cold PBS. Next, the cells were incubated with 1% BSA for 30 min and then with antibodies against LC3B (ab192890, abcam, Cambridge, UK) and P62 (ab109012, abcam, Cambridge, UK) overnight at 4 °C. Then, the cells were incubated with Cy3-labeled goat anti-rabbit IgG (H + L) (A0516, Biyuntian, China) for 1 h at room temperature in the dark. Finally, the cells were incubated with DAPI for 5 min, and then the coverslips were visualized under fluorescence microscopy (BX51TRF, OLYMPUS, Japan). The experiment was repeated three times, and five fields were randomly selected to analyze the fluorescence intensity using ImagJ-5.0 software (windows, 64-bit Java 1.8.0_112).

### Transmission electron microscopy (TEM)

The cells were harvested, fixed with 4% gluteraldehyde overnight and then postfixed with 2.5% osmium tetroxide. After dehydration in an ascending series of ethanol, the samples were embedded in paraffin and cut into 80-nm sections. Autophagy, morphology and quantity in the sections were examined by transmission electron microscopy (Hitachi-7500, Hitachi Limited, Japan). The number of autophagosomes was calculated by randomly selecting 10 cells in each group using the following formula: autophagosome density = autophagosome number/cell number.

### Flow cytometry assay

During logarithmic growth, the cells were digested with 0.25% trypsin and washed twice with PBS. Then, the cell concentration was adjusted to 1 × 10^6^ cells/ml with PBS. Next, 200 μl of Annexin V-FITC staining solution was added to a 1-ml cell suspension and incubated at room temperature for 15 min in the dark. Then, 200 μl of propidium iodide staining solution was added and mixed. Apoptosis was detected by flow cytometry (FACSVantage SE, BD, USA). The apoptotic index was calculated as follows: (number of apoptotic cells / total number of cells tested) × 100%.

### TUNEL assay

The TUNEL assay was performed using the in situ cell death detection kit-POD (TUNEL, Roche, Switzerland) according to the manufacturer’s instructions. Cells were cultured on glass coverslips overnight. After being fixed with 4% paraformaldehyde for 1 h, the cells were washed twice with PBS. Next, the cells were blocked with freshly prepared 3% H_2_O_2_ dissolved in methanol for 10 min and then washed with PBS. Then, the cells were permeated with freshly prepared 0.1% Triton X-100 at 4 °C for 2 min. After a second round of washes, the slides were then incubated with 50 μl of freshly prepared TUNEL reaction mixture for 1 h at 37 °C in a moist chamber. After being washed twice with PBS, the slices were observed under the fluorescence microscope (BX51TRF, OLYMPUS, Japan). The experiment was repeated three times, and five independent fields were randomly selected to calculate the cell numbers using ImagJ-5.0 software (windows, 64-bit Java 1.8.0_112). The TUNEL-positive rate was calculated as follows: TUNEL-positive cell number / total cell number × 100%.

### MTT assay

Cell proliferation was measured using the Cell Proliferation Kit I (MTT) (11465007001, Roche, Switzerland) according to the manufacturer’s instructions. First, 1000 cells/100 μl of cell suspension were added into each well in a 96-well plate, and 3 replicate wells were tested in each group. Then, the plates were placed in an incubator overnight (37 °C, 5% CO_2_). Then, 20 μl of MTT solution was added to each well at 24, 48, 72, 96 h, and incubated for 4 h. After removing the MTT-containing medium, 150 μl of DMSO was added. The absorbance at 490 nm was measured with a microplate reader (Gene Company Limited, Hong Kong, China). Cell growth curves were generated according to time and absorbance.

### Quantitative real-time polymerase chain reaction (RT-qPCR) assay

Total RNA was extracted from tissues or cells with RNAiso Plus (9180, Takara, Japan) according to the instructions, and the OA value of the RNA at 260 nm and 280 nm was measured by Nanodrop ND 2000 (Thermo Scientific) to calculate the RNA concentration and purity. The total RNA was reverse-transcribed into complementary DNA (cDNA) using The PrimeScript RT reagent Kit gDNAEraser (Perfect Real Time) (RR047A, Takara, Japan). Real-time PCR was performed using 2 × SYBR Premix Ex TaqTMII according to the manufacturer’s instructions. The primers used to amplify *CASC9, LC3B, P62, BCL-2, BAX* and the housekeeping gene *GAPDH* were designed using the Oligo7.0 software, and the sequences are shown in Supplementary Table. S3. The total volume of the reaction solution was 25 μl. A C-1000™ Thermal Cycler (Bio-Rad, CA, USA) was used for qPCR. The PCR amplification was performed for 40 cycles of 95 °C for 90 s, 95 °C for 10 s, and 60 °C for 30 s. The relative expression level of each gene was calculated using the 2^−ΔΔCt^ method.

### Western blotting

Cells were collected and lysed in ice-cold RIPA lysis buffer (P0013B, Beyotime, Shanghai, China) containing PMSF and phosphatase inhibitor for 30 min. The supernatant was collected after centrifugation at 14000 × *g* for 5 min. The protein concentration was determined using a BCA protein quantification kit (P0010, Beyotime, Shanghai, China). Then, 30 μg of total protein was separated on an 8 to 15% SDS-PAGE gel, and the protein in the gel was transferred to a 0.45-µm PVDF membrane. The PVDF membrane was blocked by immersion in 5% skim milk for 1 h. Next, the membrane was incubated with primary antibodies against AKT, p-AKT, mTOR, p-mTOR, LC3B, P62, BCL-2, BAX and GAPDH overnight at 4 °C. The details of the antibodies used are shown in Supplementary. Table S4. Then, the membrane was incubated with a horseradish peroxidase (HRP)-labeled secondary antibody was at 37 °C for 40 min. An ECL-Advance Western Blot Detection system (Bio-Rad) with an enhanced chemiluminescent substrate (34577, thermo scientific, USA) was used for the detection of the protein bands. The intensities of GAPDH were used as a control for all other bands. Each test was performed on the same membrane and repeated 3 times. Data were analyzed using ImagJ-5.0 software (windows, 64-bit Java 1.8.0_112).

### In vivo tumorigenicity assay

Ten specific pathogen-free (SPF) BALB/c nu/nu female nude mice (18 to 22 g, 4–6 weeks old) (Chongqing Medical University Laboratory Animal Research Institute) were randomly divided into the sh-*CASC9* group and sh -NC group with 5 mice in each group. Then, 0.2 ml of sh-*CASC9* and sh-NC SCC15 cell suspensions with a cell concentration of 5 × 10^7^ cells/ml were injected into the left back of each mouse. The tumor size was observed and recorded every 4 days for 4 weeks. After tumor formation was obvious, the nude mice were sacrificed by cervical dislocation. The tumor weight was measured using an electronic balance (A250, Denver Instrument, USA), and the maximum long diameter (*a*) and minimum short diameter (*b*) of the tumor were measured with a Vernier caliper. The tumor volume (*V*) was calculated with the following formula: *V* = 0.5 × *a* × *b*^2^. The mRNA expression level of *LC3B, P62, BCL-2* and *BAX* in the tumor tissues was detected by RT-qPCR. All animal experimental procedures were approved by the Laboratory Animal Use Management Committee of the Experimental Animal Institute of Chongqing Medical University (Approval number: 2018–040).

### Statistical analysis

The statistical analysis was performed using GraphPad Prism 7.0 (Graphpad Software, La Jolla, CA) and SPSS 23 (IBM, SPSS, Chicago, IL, USA). The Chi-Squared test was used to analyze the relationship between the *CASC9*, p-AKT and LC3B expression levels and the clinicopathological characteristics. The statistical significance of survival-related factors was analyzed by the Cox regression model in the multivariate analysis. Survival curves were plotted using the Kaplan–Meier method, and the difference of overall survival time between the two groups was statistically analyzed by the log-rank test. The two-tailed Student’s *t* test was used for comparison between two independent groups, and the one-way ANOVA test was used for the comparison of three or more means. Data are shown as the mean ± SD from at least 3 independent experiments. *P* < 0.05 was considered statistically significant.

## Supplementary information

The probe sequences used for ISH

Sequences of CASC9-siRNA/shRNA interference

Primer sequences used for RT-qPCR

Antibodies used in western blotting, ISH and IHC
