# Supplementary material for: Increased expression of lncRNA CASC9 promotes tumor progression by suppressing autophagy-mediated cell apoptosis via the AKT/mTOR pathway in oral squamous cell carcinoma
Source: Cell Death Dis. 2019 Jan 17;10(2):41. doi: 10.1038/s41419-018-1280-8 (PMC6381212; doi:10.1038/s41419-018-1280-8)
Supplement: Supplementary file 1 — The probe sequences used for ISH [file 41419_2018_1280_MOESM1_ESM.docx]

**Supplementary Table S1 The probe sequences used for ISH.**

| **Probe Sequences** |
| --- |
| 5'-GAATTTGCTTTTCTGGAACATAGCCTAGCAGAACA-3' |
| 5'-TCATGGGACTCATATTACCAGTCTTCACATTTCCTTTAAA-3' |
| 5'-CTGTCCCAAGCGACATCATTTTCAACCTGCTGAAGATCTT-3' |
