# Supplementary material for: Increased expression of lncRNA CASC9 promotes tumor progression by suppressing autophagy-mediated cell apoptosis via the AKT/mTOR pathway in oral squamous cell carcinoma
Source: Cell Death Dis. 2019 Jan 17;10(2):41. doi: 10.1038/s41419-018-1280-8 (PMC6381212; doi:10.1038/s41419-018-1280-8)
Supplement: Supplementary file 2 — Sequences of CASC9-siRNA/shRNA interference [file 41419_2018_1280_MOESM2_ESM.docx]

**Supplementary Table S2 Sequences of CASC9-siRNA/shRNA interference**

| **Group** | **Sequence** |
| --- | --- |
| si-1 | 5'-GCCUGUGAUAGCAGAACAAUU-3' |
| si-2 | 5'-UUCUCCGAACGUGUCACGUUU-3' |
| si-3 | 5'-GGAAGAAUUUCCAGAGUUUUU-3' |
| si-NC | 5'-UUCUCCGAACGUGUCACGUUU -3' |
| sh-CASC9 | 5'-CCGGAAACUCUGGAAAUUCUUCCCUCGAGGGAAGAAUUUCCAGAGUUUUUUUUG-3' |
| sh-NC | 5'-CCGGACGUGACACGUUCGGAGAUUCUCGAGUUCUCCGAACGUGUCACGUUUUUUG-3' |
