# Supplementary material for: Increased expression of lncRNA CASC9 promotes tumor progression by suppressing autophagy-mediated cell apoptosis via the AKT/mTOR pathway in oral squamous cell carcinoma
Source: Cell Death Dis. 2019 Jan 17;10(2):41. doi: 10.1038/s41419-018-1280-8 (PMC6381212; doi:10.1038/s41419-018-1280-8)
Supplement: Supplementary file 3 — Primer sequences used for RT-qPCR [file 41419_2018_1280_MOESM3_ESM.docx]

**Supplementary Table S3 Primer sequences used for RT-qPCR.**

| **Gene** | **Forward (5' to 3')** | **Reverse (5' to 3')** |
| --- | --- | --- |
| CASC9 | TTGGTCAGCCACATTCATGGT | AGTGCCAATGACTCTCCAGC |
| LC3 | AAGCCGTCCTCGTCTTTCT | AAGCCGTCCTCGTCTTTCT |
| P62 | TGGAGCACGGAGGGAA | TCTGGCATCTGTAGGGACTG |
| BCL-2 | TTTGTGGAACTGTACGGCCC | TCACTTGTGGCCCAGATAGG |
| BAX | TGGCGATGAACTGGACAACA | CACGGAAGAAGACCTCTCGG |
| GAPDH | TCAAGAAGGTGGTGAAGCAGG | AGCGTCAAAGGTGGAGGAGTG |
