# Supplementary material for: Increased expression of lncRNA CASC9 promotes tumor progression by suppressing autophagy-mediated cell apoptosis via the AKT/mTOR pathway in oral squamous cell carcinoma
Source: Cell Death Dis. 2019 Jan 17;10(2):41. doi: 10.1038/s41419-018-1280-8 (PMC6381212; doi:10.1038/s41419-018-1280-8)
Supplement: Supplementary file 4 — Antibodies used in western blotting, ISH and IHC [file 41419_2018_1280_MOESM4_ESM.docx]

**Supplementary Table S4 Antibodies used in western blotting, ISH and IHC.**

| Antibody | Catalog NO. | Brand | City | Country |
| --- | --- | --- | --- | --- |
| p-AKT(ser473) | #4060 | CST | Massachusetts | America |
| AKT | #4691 | CST | Massachusetts | America |
| p-mTOR (ser2448) | #5536 | CST | Massachusetts | America |
| mTOR | #2983 | CST | Massachusetts | America |
| P62 | ab109012 | Abcam | Cambridge | Britain |
| LC3B | ab63817 | Abcam | Cambridge | Britain |
| BAX | #2772 | CST | Massachusetts | America |
| BCL-2 | #2872 | CST | Massachusetts | America |
| GAPDH | 10494-1-AP | proteintech | Chicago | America |
| HRP-conjugated Affinipure Goat Anti-Rabbit IgG(H+L) | SA00001-2 | proteintech | Chicago | America |
